# Absorbing TiO_*x*_ thin film enabling laser welding of polyurethane membranes and polyamide fibers

**DOI:** 10.1088/1468-6996/16/5/055002

**Published:** 2015-09-25

**Authors:** Martin Amberg, Alexander Haag, Raphael Storchenegger, Patrick Rupper, Frederike Lehmeier, René M Rossi, Dirk Hegemann

**Affiliations:** 1 Empa, Swiss Federal Laboratories for Materials Science and Technology, Laboratory for Advanced Fibers, Lerchenfeldstrasse 5, 9014 St.Gallen, Switzerland; 2 Empa, Swiss Federal Laboratories for Materials Science and Technology, Laboratory for Protection and Physiology, Lerchenfeldstrasse 5, 9014 St.Gallen, Switzerland; 3 Leister Technologies AG, R&D Corporate Research, Galileo-Strasse 10, 6056 Kaegiswil, Switzerland

**Keywords:** titanium suboxides, fiber coatings, reactive magnetron sputtering, laser welding, NIR absorption

## Abstract

We report on the optical properties of thin titanium suboxide (TiO_*x*_) films for applications in laser transmission welding of polymers. Non-absorbing fibers were coated with TiO_*x*_ coatings by reactive magnetron sputtering. Plasma process parameters influencing the chemical composition and morphology of the deposited thin films were investigated in order to optimize their absorption properties. Optical absorption spectroscopy showed that the oxygen content of the TiO_*x*_ coatings is the main parameter influencing the optical absorbance. Overtreatment (high power plasma input) of the fiber surface leads to high surface roughness and loss of mechanical stability of the fiber. The study shows that thin substoichiometric TiO_*x*_ films enable the welding of very thin polyurethane membranes and polyamide fibers with improved adhesion properties.

## 1. Introduction

Two common technologies, ultrasonic welding and hot plate welding, are used to join thermoplastic materials [1, 2]. A more recent, yet less common technique is laser-transmission welding, opening new possibilities and applications such as joining membranes with liquid- and gas-tight properties [3]. For medical and automotive (airbag) applications, laser welding is already established [4]. For systems where gas and liquid has to be transported, filtered and separated (osmosis), a high flow of the media has to be guaranteed. Laser welding can locally weld the supporting tissues (single fibers, open webs and interlaced yarns) to thin membranes, filters and functional structures enabling the mechanical fixation and a high permeability.

For the welding of two thermoplastic materials it is crucial to have a transparent top layer, where the wavelength of the near infrared (940 nm) laser is not absorbed. The second layer underneath needs to absorb the laser energy that converts to heat very locally. The controlled energy input increases the temperature to the melting point of the materials, which partially melt, merge and, after cooling, build a joined solid. The welds possess good seam strength of 40%–100% of the material strength. The main advantage of laser transmission welding is the focused (local) heat generation with limited heat transport through the material itself as well as ease of automation, clean and fast processing (up to 20 m min^−1^) [5].

To achieve a sufficient absorption of the laser radiation, suitable pigments, dyes or, most used, carbon black particles are added to the polymer [6–10]. These pigments are mainly incorporated before extrusion or spin-dyeing and are present throughout the whole volume of the material. Introducing dyes and pigments can be seen as impurities for the bulk material and can, to a certain extent, affect the material properties such as reducing tensile strength and increasing elongation [11]. Carbon black is widely used in two commercial laser welding products, namely a polyester membrane with 85% absorption from Sympatex (Germany) and a polyurethane membrane with 90% absorption from Fait Plast (Italy). The carbon black is solubilized with polypropylene before spin dying and therefore not suited for all thermoplastic compound combinations.

A novel approach has been investigated by the authors, where the absorbing material is applied to the top surface by reactive magnetron sputtering on multifilament (here: polyamide) fibers [12]. These thin films just cover the surface of the intended absorber material. In the case of laser welding, the infrared light is thus directly absorbed at the surface. Using the technology of applying thin films of titanium suboxide by reactive magnetron sputtering opens the possibility to control absorption properties without using carbon black. Since carbon black is manufactured by the pyrolysis of heavy fuel oils, it cannot be used in medical applications and in the manufacture of pharmaceuticals or edible inks [13]. Black titanium oxides are non-stoichiometric transition metal oxides (Ti_2_O_3_), which are promising in terms of their optical properties, chemical stability, relatively high electrical conductivity and environmental friendliness [14–16]. In comparison to TiO_2_ [17], less attention has been paid to the physics and applications of the suboxide films [18, 19]. One recent publication deals with sol–gel preparation and characterization of black titanium oxides such as Ti_2_O_3_ and Ti_3_O_5_ [15]. The disadvantage of this approach is the carbon thermal reduction reaction at 1000 °C that is not directly applicable for polymer substrates.

Therefore, this work focuses on the optical absorption properties of thin films (black coatings) of titanium suboxides for laser welding. The sputter-deposited coatings enable very thin membranes in the range of 10–50 *μ*m to be welded with polyamide fibers. Laser welding is thus opened up for membranes, and textile structures such as nonwovens, fabrics and single fibers can be combined and joined.

## 2. Experimental details

### 2.1. Titanium deposition and substrates

Thin titanium coatings were sputtered from a Ti target (diameter *d* of 5 cm, area *A* of 19.6 cm^2^, purity 99.99%, AJA International Inc., USA) on textile co-polyamid Grilon C-140 glue yarn (200 dtex f44 supplied by EMS-CHEMIE AG, Switzerland) consisting of 44 filaments of 21 *μ*m diameter each with minimum residual oil. The sputtering was performed in a lab-scale vacuum chamber (*p*_base_ = 10^−7^ mbar) using a 2″ magnetron sputtering system (‘Stiletto’ ST20-O-C-M, AJA International Inc., USA). For deposition the pressure was controlled at 2 Pa by a membrane gauge type Baratron (MKS, Germany) along with a gas flow of total 20 sccm argon with up to 2 sccm oxygen (N5 and N48, Carbagas; 10% of total flow). The magnetron was powered by a Pinnacle+generator (Advanced Energy, USA) with 100 kHz, 2 *μ*s pulse (80% duty cycle). The pulsed sputter deposition has been performed (i) to clean the target, (ii) to avoid poisoning of the target during the deposition with additional oxygen, and (iii) to achieve a smoother coating surface [20]. During plasma deposition the fiber was transported 80 times (with a spacing of 1.5 mm between the fiber windings of length 0.45 m) at a distance of 6.5 cm from the target through the coating zone. Thus, approximately 36 m of fiber is present in the plasma chamber at once enabling a reel-to-reel process for up to a kilometer of coated fiber. The speed was varied between 0.5 and 5 m min^−1^ in order to adjust the coating thickness. To reach steady state conditions and avoid the effects of target cleaning, the first 36 m of fiber had been discarded. The deposited mass of titanium on the fiber (in *μ*g per meter of fiber length) was analyzed by inductively coupled plasma optical emission spectrometry (ICP-OES; emission lines: 334.940 and 336.121 nm). The coated fibers were ashed in a silver bowl and subsequently in 1 g KOH dissolved using an open flame. The residual was solved in water and acidified with 2 ml HNO_3_.

### 2.2. Surface morphology and composition

Morphological surface investigations have been performed on fibers using optical microscopy (Keyence VHX-1000) and scanning electron microscopy (SEM, Hitachi S-4800). The chemical composition of the coatings was analyzed by x-ray photoelectron spectroscopy (XPS) measurements. A PHI 5600 LS spectrometer equipped with a non-monochromatic Mg–K*α* (300 W) x-ray source was used. Ti spectra were recorded using a pass energy of 29 eV and a step width of 0.125 eV. To sputter-clean the surface, a short (20 s) Ar ion treatment was applied *in situ* before starting the measurement. The energy scale was calibrated using the adventitious carbon signal and set to 285.0 eV. After applying a Shirley type background subtraction, the spectra of Ti were analyzed by deconvolution into eight components (four belonging to 2*p*_3/2_ and four to 2*p*_1/2_ of Ti) corresponding to different Ti oxidation states, namely TiO_2_, Ti_2_O_3_, TiO, and Ti metallic as commonly reported in the literature [18].

### 2.3. Optical properties

The optical absorbance spectra were acquired with a Lambda 19 PerkinElmer double beam spectrophotometer. It is equipped with a 200 mm integrating sphere used to measure the spectral dependence of total and diffuse reflectance over the wavelengths of 250–2500 nm. Fiber samples were measured spooled 1 mm thick on a card. The reflectance of Spectralon (LabSphere) was used as 100%. The absorption was calculated from the following formula:

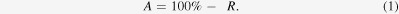



### 2.4. Laser welding

**Figure 1.**
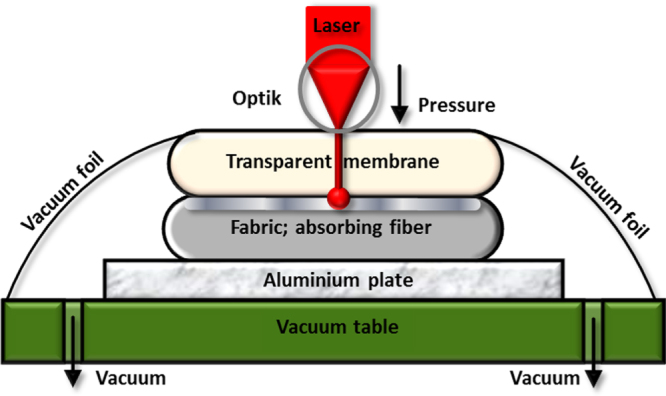
Setup of the laser welding experiment: stacking of the samples, from bottom to top, consisted of an aluminium plate above the woven sample and the membrane. The polyurethane membrane was fixed with a polyethylene foil by vacuum.

Before laser welding the treated yarn was woven into a canvas 1/1 performed on a hand loom. To maintain stability in the fabric, the coated yarn was inserted as every third weft. The other two weft and the warp were made of polyester yarn (30 tex, Trevira CS, Trevira GmbH, Bobingen, Germany). The fabric including the titanium oxide-coated yarn was welded together with a polyurethane membrane (15 *μ*m, NR: 05 white, supplied by Fait Plast S.p.A., Italy). Laser welding was performed with a laser diode emitting at a wavelength of 940 nm. A glass fiber connects the generator with a movable, air guided spherical optic (GloboOptik, Leister Technologies AG, Kägiswil, Switzerland). The optical system was installed on an X-Y vacuum table (Gunner Int., Switzerland) that prevents dislocation of the layers during the welding process. On top of the samples a high NIR transparent vacuum foil made of polyethylene was applied to accomplish the fixation of the stack of layers to the table by applying vacuum as shown in figure 1.

The sample stacks were welded by scanning the laser line by line over an area of 100 cm^2^. Laser energy of 10 W on a spot size of 3.14 mm^2^ was introduced through the optical system. The system was pressurized with 1 bar pressure, and a speed of 3 cm min^−1^ was used for laser welding.

### 2.5. Mechanical properties of fibers and welds

Tensile strength (in dependence on EN ISO 13934-2) was measured on the metalized fibers five times per sample with an Uster Tensiorapid (Uster Technologies, Switzerland) before weaving and welding. After weaving and laser welding the peel strength test (EN ISO 13934-1) was performed on a 25 mm × 50 mm sample with a Zwick Z100 device (Zwick GmbH & Co., Germany). Due to the low mechanical stability of the membrane, the membrane was stabilized with a Scotch tape. The membrane was then clamped and peeled off the woven substrate. A pretension of 1 N and a speed of 100 mm min^−1^ were set for the peel-off experiments.

## 3. Results and disscussion

### 3.1. Morphology of the deposited TiO_*x*_ coatings

**Figure 2.**
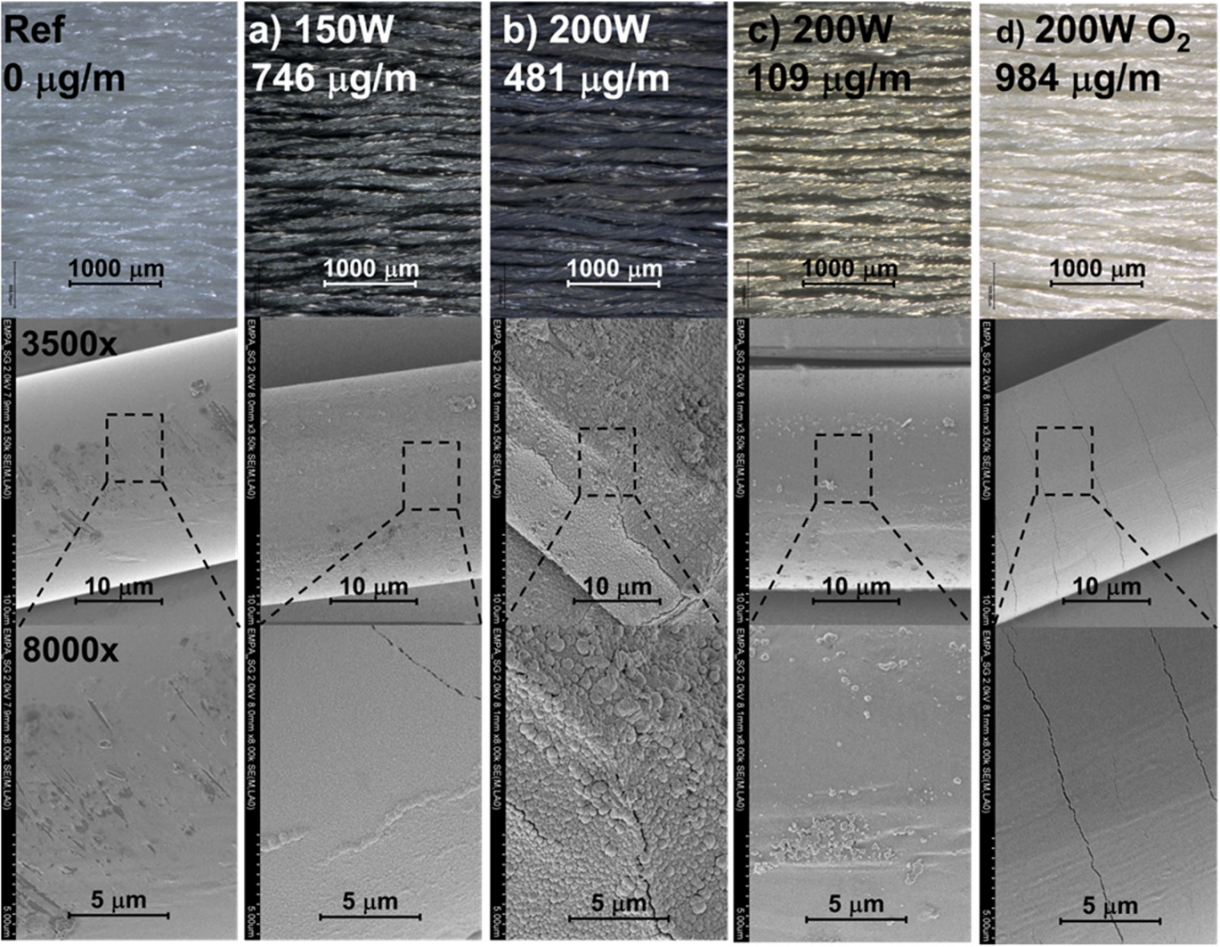
Optical appearance and surface morphology of the coated samples deposited at different power inputs (150 and 200 W) and winding speeds of 0.5 (a) and (d), 1 (b), and 5 m min^−1^ (c). For sample (d) an additional O_2_ gas flow rate of 2 sccm has been applied.

The surface of the Grilon C-140 polyamide yarn was modified by a thin layer of titanium to change its optical absorption behavior. In figure 2, titanium films sputter-deposited on yarns, using different process parameters (see table 1), are shown. The upper half shows pictures taken with the optical microscope, whereas the lower represents SEM pictures. The uncoated reference multifilament material is depicted on the left side (figure 2 labeled ‘Ref’). Figures 2(a) and (b) show the coatings resulting from a target power of 150 and 200 W, respectively. The colors of both coatings appear to be black. The coating deposited at 150 W (figure 2(a)) revealed a homogeneous and smooth surface with almost no cracks. In contrast, the same coatings deposited at 200 W (figure 2(b)) showed a rough surface with spherical particles up to 0.7 *μ*m in size (diameter). Furthermore, the fiber was flattened (not showing a round circumference). The protruding grains and the deformation of the fiber geometry indicate an overtreatment of the sample surface, in this case caused by a high heat flow from the magnetron cathode resulting in substantial substrate heating [21]. Overtreatment is first leading to melting (by reaching the glass temperature of approximately 60 °C–75 °C), followed by recrystallization and chain scission of the polymers, resulting in a loss of mechanical strength of the fiber [11, 22, 23].

**Table 1.** Summary of deposition conditions, mass deposition rate for Ti and mechanical properties.

Power @ 100 kHz, 2 *μ*s (W)	Fiber speed (m min^−1^)	O_2_-flow (sccm)	Ti mass deposition rate (mg min^−1^)	Absorption @940 nm (%)	Fiber tensile strength (N)	Peel strength (N)
Ref uncoated	—	—	—	13	5.5 ± 0.4	∗
150	0.5	0	0.38	95	4.9 ± 0.2	5.0
200	1	0	0.48	97	1.9 ± 0.1	3.7
200	5	0	0.54	88	4.9 ± 0.2	3.8
200	0.5	2	0.49	43	4.7 ± 0.1	∗

Note.‘∗’ indicate that the material is not laser weldable, no joint is built.

A very thin TiO_*x*_ coating of 109 *μ*g m^−1^ (ca. 8 nm film thickness) appeared light gray (see figure 2(c)). The thin coating was performed with the same parameters as in the process using 200 W but with a higher winding speed of 5 m min^−1^. Thereby, the film thickness and the applied heat load during the deposition were reduced. The reduced heat load also decreased the amount of spherical particles (figure 2(c)). Samples (a) and (c) thus comprise comparable morphologies with a higher deposition rate for the coating deposited at the higher power, while the deposition rate might be slightly affected by the polymer damage as obtained with sample b (see table 1). Figure 2(d) shows a deposition process at 200 W with an additional oxygen flow rate of 2 sccm resulting in a white color of the coating. These coatings with typical oxide character (hard and brittle) showed a periodic crack formation across the fiber length with an interval of 5 *μ*m. During the reactive sputtering process with increased oxygen partial pressure an increased secondary electron coefficient at the target surface (caused by the poisoning of the target) and forming O^−^ species are found in the plasma [24, 25]. These effects are observed by increased target current and simultaneous lower deposition voltage leading to reduced plasma–substrate interaction [26]. Additionally, the formed TiO_2_ surface has a high reflectivity in the range of VIS to NIR (see also section spectroscopic investigation) reflecting the radiated energy from the plasma. Nevertheless, the deposition rate is only slightly decreased (see table 1).

### 3.2. Titanium coatings and fiber tensile strength

**Figure 3.**
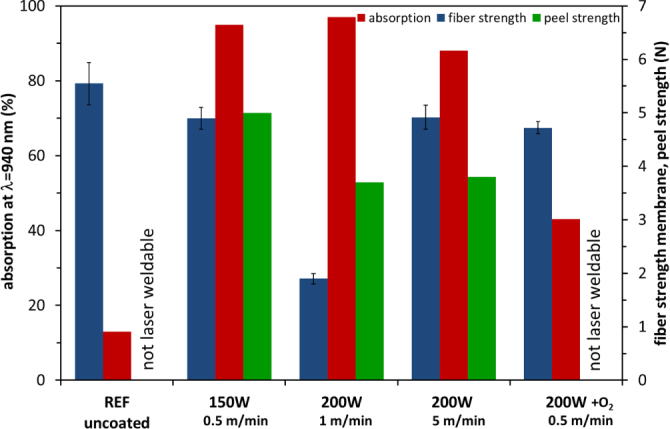
Tensile strength (error bars indicate standard deviation of the measurements), absorption of the TiO_*x*_ coated fibers and its peel strength of the laser transmission welded webs-membrane joint.

Studying tensile strength is the easiest way to measure the change in a polymeric structure. To proof the statement that was made by the microscopic analysis, tensile tests have been performed. The results of these tests are shown in figure 3 exhibiting the lowest tensile strength value for the yarn coated at 200 W and a speed of 1 m min^−1^. This finding is congruent with the optical microscope analysis, indicating the intrinsic damage to the fiber through the 200 W plasma process (performed at the lower speed of 1 m min^−1^). All other samples show minor mechanical losses of about 10%.

### 3.3. Influence of oxygen during the sputter deposition

**Figure 4.**
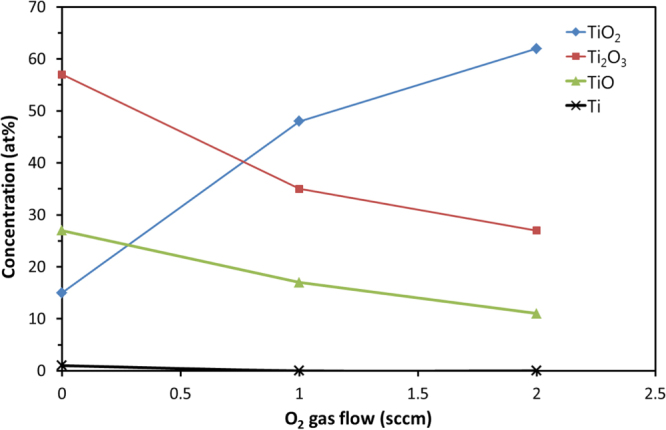
XPS analyses of the titanium and its oxides prepared by pulsed magnetron deposition as a function of additional oxygen gas flow.

The different Ti oxidation states of the fiber coating were studied by XPS and the results are shown in figure 4. The titanium coatings deposited without additional oxygen were found to be not in a pure metallic state. A predominance of TiO_2_ and sub-stoichiometric oxides (Ti_2_O_3_, TiO) were found caused by oxygen impurities and subsequent natural oxidation (passivation). A minor oxidation effect might also be attributed to outgassing of water vapor from the fiber during the coating process [27]. However, the oxidation was found to be much lower for the metallic sputtering (yielding the formation of black Ti suboxides) as compared to small O_2_ additions. Recent observations made on reactive gas gradients for combinatorial reactive magnetron sputtering confirm the existence of black coatings [28]. It was shown that these coatings have a mixture of cubic TiO, amorphous and nanocrystalline TiO_*x*_ (*x* < 1).

For coatings with additional oxygen at an O_2_/Ar flow ratio of 5% (1 sccm) of total flow, the sputtering process turned into the transition mode of reactive sputtering and the TiO and Ti_2_O_3_ concentrations decreased from 27% to 17% and from 57% to 35%, respectively. The TiO_2_ concentration increased, whereas no metallic Ti is detected. Another increase of oxygen to a ratio of 10% (2 sccm) further enhanced the TiO_2_ concentration corresponding to a decreased fraction of the suboxides in the thin film resulting in white ceramic-like coatings.

### 3.4. Optical absorption

**Figure 5.**
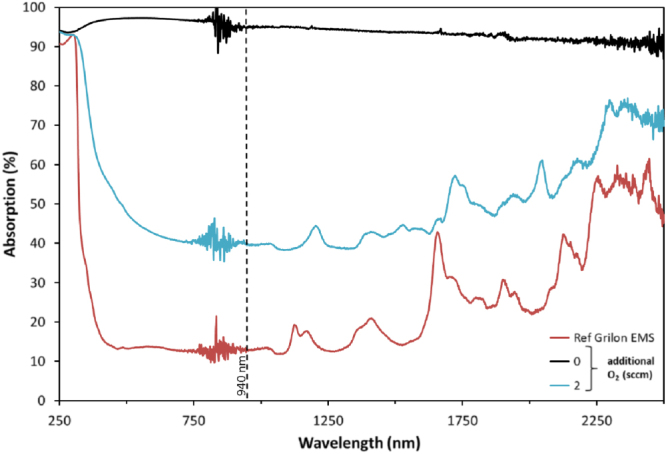
Optical spectra of TiO_*x*_ coated fibers with and without additional oxygen gas flow during deposition. The uncoated fiber is shown as reference. The dotted line indicates the wavelength of the laser used in the welding process.

As can be seen from figure 2, the O_2_ flow rate has a remarkable influence on the color of the coating. To investigate the NIR range, photo-spectroscopic measurements have been performed on the TiO_*x*_ coatings. Figure 5 depicts photometric spectra from 250 to 2500 nm and the impact of the O_2_ gas flow rate during sputter deposition. The dashed line represents the NIR laser operating wavelength of 940 nm. The untreated fiber has a very low NIR absorbance (∼13% at wavelength of 940 nm) and is not laser-weldable as is discussed below. The coated fibrous sample leads to an increase of NIR absorbance which is preferable for laser welding. Coatings with a high oxygen content (e.g. >60% TiO_2_ as in the case of using 2 sccm O_2_) reveal a drop in the NIR absorption, which agrees with the results of Levinson *et al* [29]. As a side note, it can be mentioned that all Ti-oxide coatings exhibit absorption in the ultraviolet light.

### 3.5. Peel strength test of the laser welded parts

**Figure 6.**
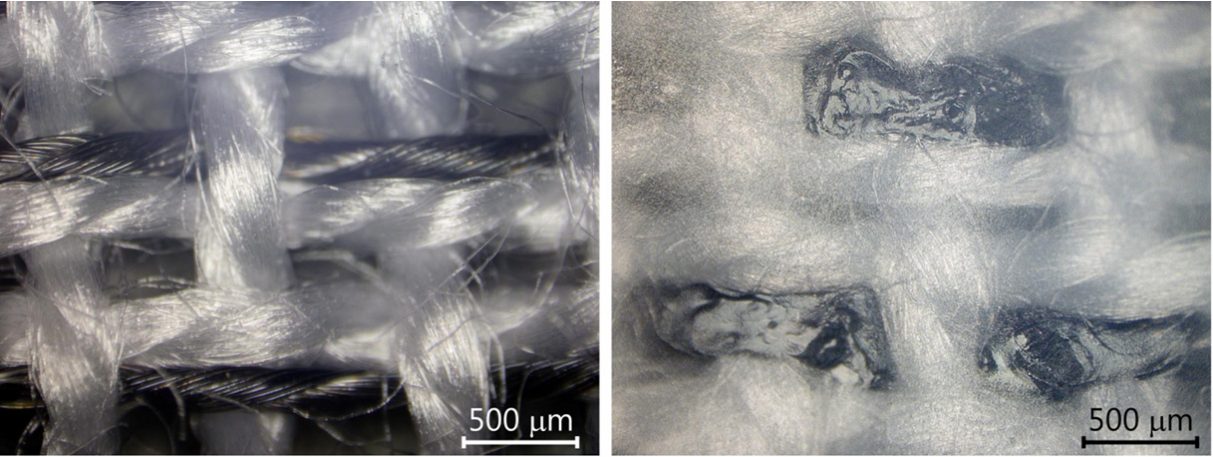
Top view of the fabric (canvas 1/1) before (left) and after laser welding (right) at 150 W through the transparent polyurethane membrane. The picture on the right appears diffuse because it was taken through the transparent membrane.

To investigate the adhesion of the membrane to the fabric after laser welding, the fiber samples were first woven to a fabric as shown in figure 6 on the left. The Ti coated yarn is visible every third weft yarn appearing dark beside the white (uncoated) polyester yarn. After weaving the fabric was covered with a NIR transparent membrane and subsequently laser welded. The laser welded web is shown in figure 6 on the right. The polyurethane membrane on top causes the diffuse appearance of the picture. The welding points are locally joining the polyurethane membrane to the Ti coated fiber and leaving the uncoated polyester yarn unaffected.

After laser welding the membrane was peeled off the fabric by measuring the peel strength. The absorption results and the peel strength are summarized in figure 3. Laser welding could not be performed with the uncoated fiber due to low absorption values. Coatings using depositions with 150 W (without O_2_ addition) yielding high titanium suboxide concentrations (mainly Ti_2_O_3_ (57 at%) and TiO (27 at%)) offered a high absorption value (>90%) enabling laser welding resulting in a high peel strength of 5 N. Higher deposition power input (200 W) caused damages in the polymeric structure (as discussed before) and therefore, resulted in decreased mechanical properties, even though the coating had a high NIR absorption. Films with a lower coating thickness processed at a higher speed of 5 m min^−1^ show a corresponding drop in the absorption but are still laser-weldable. The reduced absorption leads to lower heating efficiency and has to be compensated by higher laser power during laser welding. At constant laser power, as is used here, lower absorption values also lead to a reduction of the peel strength. The fiber with a high TiO_2_ concentration is not laser-weldable, due to its low NIR absorption of the film.

## 4. Conclusions

Thin, black titanium suboxide coatings deposited by reactive magnetron sputtering on polyamide fibers were investigated for laser welding application. Black titanium suboxide (Ti_2_O_3_ and TiO) coatings could be observed without addition of oxygen, whereas admixture of O_2_) in the argon-based sputtering process increased the ceramic character of the coatings showing enhanced TiO_2_ content. The added oxygen turned the process reactive, while the target stayed in the transition mode as indicated by comparable deposition rates. The coatings and its chemical oxygen content were analyzed by XPS. Films produced with 150 W sputtering power showing high TiO and Ti_2_O_3_ (e.g. low Ti and TiO_2_) concentrations were found to improve the absorption of NIR wavelength up to 97%. This absorption value is significant higher than those for commercial carbon black containing products (85% for polyester membranes and 90% for polyurethane). The observed high absorption of the applied TiO_*x*_ layer enables the local laser heating on the surface with already very thin coatings of 110 *μ*g m^−1^ (ca. 8 nm film thicknesses) deposited TiO_*x*_ revealing 88% absorption. The subsequently performed laser welding experiments of the 15 *μ*m thick membrane to the carrier web revealed good mechanical performance in the range of 5 N peel strength (for TiO_*x*_ coatings of ca. 50 nm thickness as deposited at 150 W power). Treatments at 200 W led to a heat excess on the fiber surface and structural damages of the polymer material. Therefore, the polymer fiber lost its mechanical properties, and a reduction in tensile strength for the fiber as well as low peel strengths for the welded parts was observed. Additional O_2_ gas flow (2 sccm) formed mostly TiO_2_ which has a lower NIR absorption of 40%. Therefore, as-coated fibers are not laser-weldable.

The peel strength of the welded fabrics on membranes is associated with the investigated NIR absorption. Absorption values of higher than 90% showing optimized peel strength of 5 N between the membrane and the fabric. Thin films of titanium suboxides on single fibers enable a local laser transmission sintering of thin membranes. Owing to the high efficiency even at nanometer thickness, thin films of TiO_*x*_ suboxide can also be applied to flexible materials such as fibers (as shown here), membranes and thin foils.
